# A Theory of Mind investigation into the appreciation of visual jokes in schizophrenia

**DOI:** 10.1186/1471-244X-5-12

**Published:** 2005-02-24

**Authors:** Dominic Marjoram, Howard Tansley, Patrick Miller, Donald MacIntyre, David G Cunningham Owens, Eve C Johnstone, Stephen Lawrie

**Affiliations:** 1 Department of Psychiatry, University of Edinburgh, Edinburgh, UK; 2 University of Edinburgh Medical School, Edinburgh, UK

## Abstract

**Background:**

There is evidence that groups of people with schizophrenia have deficits in Theory of Mind (ToM) capabilities. Previous studies have found these to be linked to psychotic symptoms (or psychotic symptom severity) particularly the presence of delusions and hallucinations.

**Methods:**

A visual joke ToM paradigm was employed where subjects were asked to describe two types of cartoon images, those of a purely Physical nature and those requiring inferences of mental states for interpretation, and to grade them for humour and difficulty. Twenty individuals with a DSM-lV diagnosis of schizophrenia and 20 healthy matched controls were studied. Severity of current psychopathology was measured using the Krawiecka standardized scale of psychotic symptoms. IQ was estimated using the Ammons and Ammons quick test.

**Results:**

Individuals with schizophrenia performed significantly worse than controls in both conditions, this difference being most marked in the ToM condition. No relationship was found for poor ToM performance and psychotic positive symptomatology, specifically delusions and hallucinations.

**Conclusion:**

There was evidence for a compromised ToM capability in the schizophrenia group on this visual joke task. In this instance this could not be linked to particular symptomatology.

## Background

### Theory of Mind and schizophrenia

Theory of Mind (ToM) describes the ability to recognise that other people have minds containing beliefs and intentions and to be able to interpret these correctly. The term, first coined by Premack and Woodruff [1], is also referred to as mind-reading [2] or 'mentalising', when the correct inferences regarding the intentions and belief of others are used to predict and control behaviour [3].

ToM ability has been conceived as a capacity to represent epistemic mental states comprising an agent and an attitude to the truth of a proposition e.g. "Peter believes that it is raining" [4,5]. The truth of this proposition concerning the mental state of an agent (Peter, who believes it is raining) need not be affected by the truth of the embedded proposition (it is raining), which may be false [6,7]. In this way, Leslie and Roth [6] proposed that a major requirement for computing such representations is a mechanism that decouples the content of the proposition (it is raining) from reality. These special representations have come to be termed metarepresentations or M-representations.

It is widely reported that there are observed ToM deficits in schizophrenia from the numerous behavioural and neuroimaging studies that have been conducted investigating this phenomenon [e.g. [8]]. It is proposed that certain symptoms characteristic of schizophrenia may also reflect specific impairments in ToM abilities [see [9]], these being positive symptoms of delusions and hallucinations and chronic negative symptoms. Frith [10] also hypothesised that positive schizophrenic symptoms could result from impairment in metarepresentation. In more detail, Frith hypothesized that in certain cases of schizophrenia something may go wrong with the decoupling process involved in computing metarepresentations [11]. This might occur in two ways. Using the above scenario, firstly the content (it is raining) becomes detached from the rest of the proposition (Peter believes that...) and secondly, the content is perceived as a representation of the real world rather than someone's belief about it. This statement, unattached to any implication that it is a thought or belief of the patient, or another person, may then be misconstrued, e.g. as a third-person auditory hallucination. Different forms of hallucination may be experienced according to the precise propositions misperceived. Misinterpretation of the behaviour or intentions of others may manifest as the delusions of reference, misidentification and persecution, experienced by some individuals with schizophrenia. Indeed, it can be said that rather than an absence of ToM capabilities in these individuals there is actually an inappropriate and excessive use of basically intact theory of mind capabilities [9]. This follows since a basically intact theory of mind mechanism is needed, say, to infer other people's persecutory intentions (even when these are mistaken inferences) and there is an over-attribution of intentions of this type in persecutory-deluded people with schizophrenia [12,13]. Frith has also referred to a distinction between over-mentalising in schizophrenia and under-mentalising in autism [14].

### Pictorial studies

Sarfati et al used a strictly pictorial task in which 3 picture cartoon sequences were shown depicting a character producing an action and the participants had to choose the fourth and final picture from a choice of three images [15]. Successful image choice depended on the understanding of the character's intent behind the action. They found that individuals with schizophrenia who had thought and speech disorganisation had a significant specific difficulty attributing mental states to others.

Sarfati et al then enlarged this experimental protocol by introducing a verbal dimension to the task [16]. There were now two answer conditions to the original 3 picture cartoon sequences relaying character intent: the pictorial condition identical to the above and a new verbal condition where the choice of endings were comprised of verbal sentences. Disorganised individuals with schizophrenia performed significantly worse than the other experimental groups. Interestingly, all the groups' performance improved in the verbal condition, but the presence of verbal material did not make the disorganised patient's performance similar to that of the other groups. Sarfati et al followed this work up by looking at the difference in performance on the same task before and after the introduction of the verbal answer condition [17]. They compared a schizophrenia group and a matched control group. The entire control group and half the schizophrenia group who did not perform at the best level in the pictorial answer condition, remediated with verbalization. In contrast to their a priori hypothesis, it was not schizophrenia patients with thought and language disorders who remediated in the verbal condition.

Langdon et al, used a task comprised of 4 card black and white cartoon picture sequences of four varieties: social script stories testing logical reasoning about people without needing to infer mental states, mechanical stories testing Physical cause and effect reasoning, false belief stories testing general mind reading abilities and capture stories testing inhibitory control. Cards were place face down in a square layout and participants had to turn the cards over and place them in the correct order to show a logical sequence of events. In order to control for possible contributory effects of executive dysfunction, inhibitory control was tested using capture picture-sequences and executive planning was tested using the Tower of London task.

In both studies, it was found that individuals with schizophrenia showed a selective ToM impairment which could not be completely explained by reasoning, planning deficits or poor inhibitory control [18,19].

Brüne showed individuals a muddled cartoon 4 picture sequence depicting a ToM scenario between characters [12]. The participants had to put the pictures into the correct sequence and then answer first and second order ToM questions related to the depiction. Where as first-order questions require acknowledgement of what one story character thinks about the world, second-order questions require acknowledgement of what one story character thinks about another story character's thoughts. The schizophrenia group was outperformed by the control group.

Corcoran et al used visual jokes to look at potential ToM deficits in schizophrenia [3]. Two sets of jokes were used: a Physical set of slapstick humour that did not require ToM capabilities to understand the joke contained within the picture and a ToM set in which an appreciation of the mental states of the characters (false belief and deception) were required. ToM deficits were found in individuals with schizophrenia exhibiting passivity phenomena (e.g. thought insertion/withdrawal) and behavioural disorders.

The primary interest of the current study was to examine the associations between specific schizophrenic symptoms and ToM capabilities using the cartoon method devised by Corcoran [3], but with a larger battery of visual jokes (over treble the number of picture stimuli). Patients with schizophrenia were compared to a closely matched group of healthy controls. It was anticipated, in keeping with Frith's model [11], and the data from Corcoran et al [3], that not only would the schizophrenia group perform significantly worse than the controls, but that the severity of positive symptoms, in particular hallucinations and delusions, would be most strongly associated with ToM impairment.

## Methods

### Participants

Forty participants aged from 19–65 years were recruited for this study. Twenty of these had a diagnosis of DSM IV schizophrenia [20]. These were either in-patients of an acute psychiatric ward who were clinically stable and awaiting discharge, or outpatients attending clinics at the Royal Edinburgh Hospital. They were all receiving antipsychotic medication. Antipsychotic medication dose at time of testing was recorded for each patient and using standard published tables was converted into daily chlorpromazine equivalent dosage [21,22]. Twenty healthy volunteers from various community and hospital sources were also recruited as a control group. An estimate of their current level of overall intellectual function was made using the Quick Test [23]. Demographic characteristics for both the experimental and control groups are shown in Table 1 and the clinical details of psychiatric participants can be seen in Table 2.

**Table 1:** The demographic characteristics-mean (SD) – of the subject groups

Group	*n *(m:f)	Age	Estimated IQ	Years of Education
Schizophrenia	20 (12:8)	39.8 (11.6)	97 (9.5)	13.3 (2.9)
Control	20 (11:9)	39.8 (13.2)	100 (7.7)	13.5 (2.5)

**Table 2:** Clinical details of the patients with schizophrenia

Age of onset	Duration of illness(yrs)	Number of admissions	Medication	
Mean (sd)	Mean (sd)	Mean (sd)	Typical	Atypical
			Antipsychotics	
28.4 (10.6)	10.9 (11)	8.85 (13.2)	40%	60%

### Symptom assessment

To assess their present symptomatology, the schizophrenia patients were assessed on the Krawiecka Standardized Scale for Rating Chronic Psychotic Patients [24]. Symptoms present over the previous week, or signs at interview, are assigned a score on a five-point scale (where 0 = absent, 1 = mild, 2 = moderate, 3 = marked, 4 = severe). Ratings are given for four positive symptoms (coherently expressed delusions, hallucinations, incoherence and irrelevance of speech and incongruity), two negative symptoms (poverty of speech and flattened behaviour) and three non specific symptoms (depression, anxiety and psychomotor retardation). As a result, the maximum scores obtainable were 16 for positive symptoms, 8 for negative symptoms and 12 for non specific symptoms. The Krawiecka scores were also used to investigate in more detail the effect specific positive symptomatology had on ToM capabilities: the scores out of four given for delusions and hallucinations were used in this analysis.

All participants in this study gave written, informed consent.

### The task

**Figure 1:**
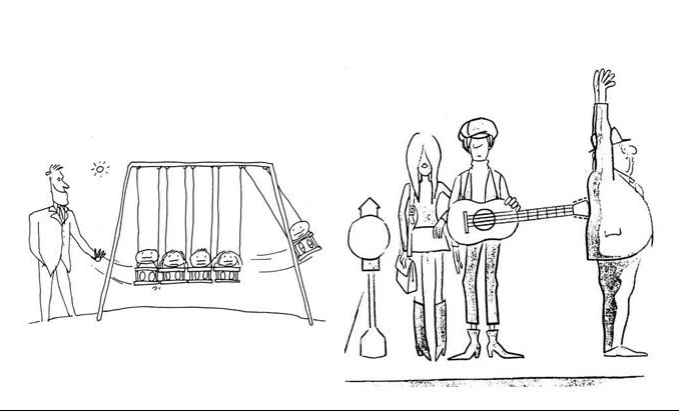
(a) An example of the Physical jokes subset. (b) An example of the ToM jokes subset.

Sixty-three single-image cartoon jokes, printed on A4 cards were generously provided by the authors of previous studies [25]. Thirty-one of these were designated to be 'theory of mind cartoons'. Understanding the humour in these jokes required the attribution of ignorance, false belief or deception to one of its characters and therefore, an analysis of their mental state. The other 32 jokes were Physical ("slapstick") or behavioural in nature and subsequently did not require ToM capabilities for their correct interpretation. All of the images were caption-less. Examples of each type are shown in Figure 1.

It was explained to the subjects that they would be shown cartoons intended to be funny. The two complete sets of cartoons were then shown to each subject in turn. The order in which they were presented was alternated so that half the participants viewed the ToM cartoons first, and half viewed the ToM cartoons second.

The subjects were shown each joke one by one and instructed to indicate to the observer when they believed they had understood its meaning. This response time was then recorded to the nearest second using a stopwatch. The participants then gave a short explanation of their interpretation of the joke's meaning. Responses were scored 1 for a correct answer and 0 for an incorrect answer. For a theory of mind answer to be correct, appropriate mental state language had to be used. Furthermore, participants were asked to subjectively grade each cartoon image for humour and difficulty on a scale of 1–5, where 1 was not funny or very easy and 5 very funny or very difficult respectively.

Simple Physical descriptions of the scenario were required for the Physical joke responses to be scored correct. An example of acceptable responses can be viewed in Table 3.

**Table 3:** Examples of acceptable and unacceptable replies to jokes featured in Fig 1

(a) Physical Joke
*Acceptable responses*
'The man is using the swing like a giant Newton's Cradle'
'The children are swinging against each other, like one of those desk toys'
*Unacceptable responses*
'The man is happy because the children are on swings'
'The man wants to send him on the end flying off the swing, so he gets hurt'

(b)*Theory of mind joke*
'The man thinks that someone is putting a gun in his back, but it's a guitar'
'The couple don't realise that they are making the man think he is being robbed'
*Unacceptable responses*
'The couple are waiting for a bus and the man is jumping to reach something'
'The couple are trying to push the man over with the guitar so that they can get on the bus first'

Tests were all performed in quiet, distraction-free rooms.

### Statistical analysis

Data analysis was performed using SPSS for Windows Version 11.0.

General linear model repeated measures ANOVA was used to determine the significance of any difference in the Physical versus ToM scores seen between the groups. General linear model ANCOVA controlling for Physical joke score was used to investigate the selectivity of any group difference in ToM capabilities. Linear regression analysis was used to relate Physical and ToM scores to Krawiecka sub-totals for positive, negative and non specific symptoms, individual Krawiecka symptoms, medication dose and joke block presentation order. Independent two-tailed t-tests were used to compare the group score differences in the two conditions (when carrying out simple contrasts following the general linear model repeated measures ANOVA), the average subjective ratings for humour and difficulty assigned to the stimuli by the participants, and the average response times to get the jokes.

## Results

### Patients with schizophrenia compared to controls

Using general linear model, repeated measures ANOVA, highly significant main effects were found for repeated measure (i.e. joke type: F = 112.9, p < 0.0001) and group (F = 42.6, p < 0.0001) as well as a significant interaction of group by joke (F = 10.3, p = 0.003). Table 4 summarises this.

**Table 4:** Performance on Physical and ToM jokes between the study groups

	Physical jokes score mean (sd)	ToM jokes score mean (sd)
Schizophrenia Group	23.3(4.5)	12.7(6.2)
Controls	28.2(2.94)	22.6(2.4)

Follow-up t-tests comparing individuals with schizophrenia to controls were highly significant for both the ToM condition (p < 0.0001) and the Physical condition (p < 0.001).

Additionally, within both the patient and control groups, scores were significantly worse for ToM jokes than Physical jokes (p < 0.0001 for both groups). However, the significant interaction showed that the difference of 10.6 for the patient group was greater than that for the controls (5.6). Using the general linear model, ANCOVA, controlling for Physical joke score, a significant group difference on ToM joke scores was still evident, F = 19.5, p < 0.05.

The two groups were well matched for age, IQ and sex, and any difference between them was shown to be insignificant by independent 2-tailed t-test (p > 0.1). It was unnecessary, therefore, to perform regression analyses to co-vary for these factors.

### Subjective joke ratings and response times and order of joke set presentation

It was found via independent T-test analysis that there was no significant difference between the schizophrenia patients and control participants' subjective ratings for humour and difficulty or between the average response times of correct responses (p > 0.05). Results are summarized in Table 5.

**Table 5:** Subjectivity scores and response times

	Picture Condition	Average humour score	Average difficulty score	Average time for correct responses
Controls	Physical	2.3 (.48)	1.9 (.62)	5.04 (2.2)
	ToM	2.4 (0.35)	1.9 (.57)	5.2 (2.9)

Schizophrenia Group	Physical	2.4 (.47)	2.4 (.68)	7.2 (2.5)
	ToM	2.6 (0.42)	2.4 (.66)	6.8 (2.7)

NB: Values are means; standard deviations in parentheses.

Furthermore, linear regression indicated that the order of presentation of the joke sets had no significant effect on ToM or Physical joke scores.

### Symptoms

Correlations were run to investigate the relationships between performances on ToM and Physical jokes and different symptom scores (assessed on the Krawiecka five-point scale). These data are displayed in Table 6.

**Table 6:** Krawiecka symptom scores in patients with schizophrenia and their association with performance on ToM and Physical joke conditions.

	N	Mean Krawiecka Score	SD	Correlation with ToM*	Correlation with Physical*
Positive symptoms	20	5.0	3.2	-0.029	0.36
Negative Symptoms	20	1.6	1.8	-0.108	0.015
Non specific Symptoms	20	1.6	1.8	0.100	0.157
Delusions	20	2.5	1.6	0.153	-0.083
Hallucination	20	1.9	1.7	-0.053	0.173
Depression	20	0.65	0.875	0.306	0.222
Incoherence of Speech	20	0.3	0.657	-0.194	0.097
Poverty of Speech	20	0.45	0.826	-0.186	-0.102

* None of these correlations reach significance

As stated, performance was not significantly reduced in association with increasing severity of positive or negative symptoms as a whole or delusions and hallucinations specifically.

The features of depression, incoherence and poverty of speech were also analysed to see if they could be having an effect on the patients ToM and Physical joke performance but there were no significant findings.

The converted equivalent daily chlorpromazine patient medication doses were correlated to performance and also found to be non significant for both cartoon conditions.

## Discussion

### Schizophrenia subjects compared to controls

This study showed that individuals with schizophrenia and normal IQ had a poorer understanding of both types of jokes (and at least a reduced ability to relay their humorous intent) than matched healthy controls. This is to be expected, as schizophrenia patients have previously been reported to show poor appreciation of humour [3]. It seems unlikely that this is explained by depression as regression analysis showed it not to be significantly related to poor ToM performance.

However, the difference between the Physical and ToM joke scores was significantly greater for schizophrenia patients, than controls. This implies that it is some aspect of the schizophrenia disease process that is associated with ToM impairment in the patient group, rather than a general difficulty with appreciation of humour.

If the schizophrenia group had a poorer understanding of the jokes then we would expect this to be reflected in the subjective gradings for humour and difficulty. As shown in table 5, the schizophrenia group actually graded the jokes non-significantly higher for both humour and difficulty. Furthermore, despite both groups performing significantly worse in the ToM condition than in the Physical condition, they both graded the two joke sets as equally difficult. Possible explanations for this could be that people were instructed that the cartoons were meant to be funny and so consequently may have stated that a joke was humorous even if they didn't find a joke funny. The subjective gradings of the jokes did not necessarily require a correct understanding of the joke for a numerical value for humour and difficulty to be assigned. Everyone could give numerical gradings for a joke but not everyone could correctly describe the jokes or use the relevant mentalising language in their joke description.

It was found that both groups found the ToM jokes significantly more difficult than the Physical ones. The former were certainly more detailed and by their very nature were comprised of characters in ToM scenarios. It could be that these jokes were more difficult to understand, but there was no significant difference between the response times of the two joke types for either group. Poor verbal report of mentalistic terms may be an intrinsic feature of schizophrenia and this could have resulted in this schizophrenia group's poor performance on this set of jokes.

Language and thought are intrinsically linked and the question arises as to whether disordered verbalisation in schizophrenia is a speech disturbance only or part of a disorder in thinking [26]. Likewise, the observed ToM deficit seen in this study could reflect a lack of response in mentalistic terms, related either to a specific deficit in inferential skills or to a more general inability to verbalise others mental states [16]. As regards our patients' verbalisation skills, they all scored none or low Krawiecka scores for the symptoms of poverty of speech and incoherence/irrelevance of speech. We therefore believe that their poor performance was the result of a compromised ToM function rather than a general verbalisation expression deficit.

This data suggests that, as predicted, schizophrenia patients have problems in interpreting the thoughts of others, supporting the findings of previous work [3]. The closely matched demographic characteristics of the two groups, suggests that problems in 'mentalising' evident in schizophrenia, are not simply attributable to the influence of factors such as age, sex and, importantly, IQ.

There is however an alternative interpretation to these results. The individuals with schizophrenia may not be showing a domain -specific difficulty with ToM function but rather may be performing differentially more poorly than the control group on the more difficult ToM condition, such that the observed deficit could reflect a differential sensitivity to increased task difficulty.

### Symptom specific findings

When the obtained totals for positive Krawiecka symptoms were analysed it was found that there was not a significant relationship between higher positive symptomatology and poor ToM performance, contrary to what had been predicted. Closer scrutiny of individual positive symptoms also revealed that neither delusions, hallucinations nor speech incoherence were significantly linked to an impaired ToM performance. Previous studies have shown paranoid delusions to be significantly related to poor ToM performance, in both first and second order ToM tasks and in both verbal and pictorial paradigms [[27,28] 32]. Interestingly, Langdon et al [14], also using a pictorial paradigm, found no evidence linking poor mentalising capabilities to positive symptoms.

These findings might be attributed to several individuals who despite scoring the maximum Krawiecka score (4) for delusions, hallucinations or both, performed similarly to controls in the ToM condition.

Alternatively, perhaps the nature of our patients' delusions and hallucinations may not be those specifically implicated in ToM impairment. Unfortunately, our sample size was too small to allow further investigation of patients with different types of delusion. Unlike the findings of previous research, negative features of schizophrenia were not associated with ToM capabilities. However, the mean Krawiecka scores for these features were low within the subject group, and our number of subjects was relatively small.

### Limitations and further work

This study was limited especially for symptom sub-groups analyses, by its relatively small sample size, although we did find disease effects. With a large sample, further symptom-specific sub-groups could be made (e.g. different types of delusions or hallucinations, formal thought disorder, different aspects of negative symptomatology, etc). Furthermore, another control group of non-schizophrenia, psychiatric patients may have been useful to explore more closely the role of diagnosis as opposed to symptoms. One of our previous studies used a psychiatric control group of patients with a psychotic affective disorder and found that positive psychotic symptomatology was linked to poor ToM performance and was not diagnosis specific [29]. This implies that ToM deficits are not necessarily specific to schizophrenia but could be related to psychoses and specifically to the positive symptoms of delusions and hallucinations. Although, as acknowledged above, we found no evidence for such an association in the present study.

We believe that the Physical cartoons themselves acted as an adequate internal control. If the schizophrenia group had performed as poorly on the Physical cartoons as they did on the ToM cartoons, this could imply either a general verbalization deficit or a general cognitive impairment. Since this was not the pattern found, our results count against a domain-general interpretation of this type. Furthermore, as mentioned previously, regression analysis showed no significant effect of language impairment, as assessed using the Krawiecka symptoms of poverty of speech and incoherence of speech, on ToM joke performance. ANCOVA also showed that the group differences on the ToM jokes could not be accounted for by the group differences on the Physical jokes. This was taken as evidence for an observable and selective compromise of ToM capacity within the schizophrenia group.

However, an unrelated cognitive neuropsychological task could have been implemented testing another cognitive domain (e.g. executive function, working memory) and this could have been used to further elaborate whether the observed compromised ToM function was a specific deficit or secondary to general cognitive impairment [see for example, 18–19 who used the Tower of London task in this way].

Further research is then required in ToM and schizophrenia to see whether the presence of schizophrenia itself is enough to impair ToM capabilities or whether ToM impairment is due, instead, to presence of particular symptoms or presence of some general neuropsychological deficit. A further question that we did not address at all in the present study was whether the ToM deficits observed in schizophrenia could be state (related to fluctuating symptom severity) or trait in nature.

## Conclusion

The schizophrenia group performed significantly worse in both the Physical and ToM conditions on this visual joke task than the matched control group. The performance on the ToM condition was significantly worse and is taken as evidence for a compromised ToM capability in the schizophrenia group which is in keeping with previous research. In this instance poor ToM performance could not be significantly linked to any particular symptomatology as had been hypothesised.

## Competing interests

The author(s) declare that they have no competing interests.

## Authors' contributions

DM conceived and designed the study, collected neuropsychological test data and drafted the manuscript. HT helped implement the study and collect neuropsychological test data and co wrote first draft. DMac and DCO were involved in the psychiatric ratings of the patients and revisions of later drafts. PM advised on statistical analysis and helped to write the corresponding sections. SL supervised clinical aspects of study and revised later drafts and ECJ revised final draft and approved this version to be published.

## Pre-publication history

The pre-publication history for this paper can be accessed here:


